# Revisiting European social dialogue: A systematic literature review

**DOI:** 10.12688/openreseurope.21020.1

**Published:** 2025-10-10

**Authors:** Fernando Cárdenas Domínguez, Mercedes Fernández García, Yoan Molinero Gerbeau

**Affiliations:** 1 Instituto Universitario de Estudios sobre Migraciones, Universidad Pontificia Comillas, Madrid, Community of Madrid, 28004, Spain

**Keywords:** European social dialogue, social dialogue, industrial relations, trade unions, employers' organisations.

## Abstract

This paper conducts a systematic literature review of the European Social Dialogue (ESD), a cornerstone of participatory governance within the EU. Despite this, it is a collective bargaining tool whose scope, objectives, functioning and impact are unclear due to the various definitions and a vague and fragmented regulatory framework, which gives rise to different interpretations. Thus, this study examines its definition, regulatory framework, key actors, negotiation structures, and outcomes. ESD facilitates collaboration between trade unions, employers, and EU institutions to shape labour policies and promote social cohesion. While the mechanism has evolved through milestones such as the Maastricht and Amsterdam Treaties, its scope and effectiveness are constrained by conceptual ambiguity, fragmented regulation, and a limited capacity to enforce agreements. The research identifies challenges related to actor representation, structural coordination, and the increasing reliance on non-binding agreements. It also highlights the potential of ESD to harmonise labour standards across member states through both soft governance and regulatory tools. Proposals for improvement include clarifying the legal framework and establishing a transparency and evaluation body to monitor its impact. These findings contribute to understanding ESD's role as an evolving governance mechanism, essential for fostering equitable and adaptable labour relations in the EU.

## 1. Introduction

European Social Dialogue (hereinafter ESD) functions as a key platform within the European Union (EU), facilitating cooperation among representatives of workers, employers, and EU institutions on essential labour-related issues. By promoting a participatory governance model, ESD integrates the perspectives of trade unions and employers' associations into EU policymaking, shaping a vital framework for aligning the EU's economic integration with its social policy goals, thereby protecting labour rights and fostering fair working conditions across member states. As the EU's economic reach grows, the importance of ESD in ensuring that economic development does not deteriorate social protection has become increasingly relevant ( Iankova, 2007; Prosser & Perin, 2015).

This bargaining tool has historically progressed in tandem with the deepening of European integration. The Treaty of Rome (1957) established the foundation for enhancing labour conditions within the common market framework. This process accelerated in the 1980s under Jacques Delors, who advocated the social dimension of European integration ( García-Muñoz-Alhambra, 2022). Key milestones, such as the Single European Act (1986), the Maastricht Treaty (1992), and the Amsterdam Treaty (1997), formalised the role of ESD, empowering social partners to negotiate binding agreements under Articles 154 and 155 of the Treaty on the Functioning of the European Union (TFEU) ( De Boer *et al.*, 2005; Keller & Sörries, 1999).

Through decades, ESD has significantly shaped the EU social policy by fostering collaboration between workers and employers, ensuring their perspectives are incorporated into the legislative framework. One of its key contributions has been its crucial role in promoting social cohesion through the European Pillar of Social Rights ( Sorensen & Dumay, 2023). ESD has also been essential in addressing specific challenges in sectors such as transport and healthcare, showcasing its adaptability to meet various needs ( De Boer *et al.*, 2005). Furthermore, it has developed agreements that enhance workplace protection and well-being, showing its capacity to adapt to the evolving European labour landscape ( Zeitlin & Vanhercke, 2018). Initiatives like the Framework Agreements on Telework (2002) and Work-Related Stress (2004) exemplify how the ESD has provided tools to adapt to evolving work environments and improve occupational health ( Prosser, 2011; Martín & Visser, 2008).

Although the ESD plays a crucial role in European politics, its scope, objectives, functioning, and impact remain unclear due to widely varying definitions and a vague, fragmented regulatory framework, leading to diverse interpretations. This lack of clarity as to the functions and roles to be played by the ESD is probably due to the different positions among member states, which have resulted in the establishment of an ambiguous legal foundation under the TFEU. Consequently, not only does its implementation vary significantly across member states ( Gómez Urquijo, 2024), but it also generates a framework of uncertainty that complicates the assessment of its influence on shaping EU policies. While ESD holds significant potential as a tool for advancing 'Europeanisation'—understood as influencing domestic political, legal, and economic reforms to improve and harmonise labour standards across member states (Sedelmeier, 2011)—its lack of clarity represents a missed opportunity for effectively managing European labour relations.

Furthermore, in addition to its ethereality, the involvement of multiple actors—including trade unions, employers' associations, and EU institutions—raises questions about representativeness and legitimacy, as no comprehensive conceptual framework exists to regulate these roles, nor specific structures to negotiate their functioning. At the same time, processes of consultation and negotiation often lack transparency and consistency, which can undermine policy outcomes. Finally, discrepancies between EU-level agreements and their national implementation frequently lead to uneven labour standards and protections across the EU ( Im *et al.*, 2024).

In the current context, understanding the complex field of action of ESD requires not only examining its legal foundations but also analysing its customary functioning. This article seeks to clarify ESD's intricate and ambiguous aspects through a systematic literature review that will provide insight into its conceptualisation, regulatory framework, scope, participants, and outcomes. This review will offer a structured basis for a comprehensive understanding of this key pillar of European policy by employing the PRISMA method. Furthermore, this approach will shed light on the factors contributing to the effectiveness of the ESD, such as its role in standardising working conditions across EU member states and its capacity to adapt to evolving socioeconomic challenges. Ultimately, this contribution aims to systematise existing knowledge on this bargaining mechanism, outlining both its strengths and limitations while facilitating its examination for future research.

By synthesising insights from diverse academic perspectives, this article contributes to the ongoing discourse on ESD's relevance and limitations. It emphasises the need for a more inclusive and adaptive framework to address traditional and contemporary issues in European labour relations. Ultimately, the study aims to provide a detailed understanding of ESD's role as a key yet evolving component of the EU's social and economic governance.

The article will be structured as follows. After this introduction, it outlines the methodology used for the literature review ( Section 2). It then presents the findings, categorised into key thematic areas: definition, regulation, actors, relationships, structures, and outcomes ( Section 3). The subsequent discussion evaluates these findings in the context of ongoing debates regarding the definition and limitations of ESD and also offers some policy recommendations for improvement ( Section 4). Finally, the study concludes by highlighting the strengths and weaknesses of ESD, providing a basis for future research on this important governance tool ( Section 5).

## 2. Method

This research is based on a systematic review following the PRISMA method ( Page *et al.*, 2021). The PRISMA method structures the procedure for searching for and selecting studies, offering a multi-step screening process controlled by various reviewers, which establishes the study guidelines. This method guarantees the transparency and reproducibility of the review process, from the identification of relevant studies to the final synthesis of the results.

### Inclusion and exclusion criteria

Initially, our review aimed to include all academic texts addressing ESD. To this end, we incorporated works that explicitly mention the term, as well as those that discuss social dialogue at the European level, even if they do not directly reference the concept. To do this, articles were analysed from 1990, which is the year the first article dealt with this subject, to June 5, 2024, when the data was extracted.

We established several exclusion criteria to focus on highly relevant and high-quality studies. Studies focused solely on social dialogue within national contexts were excluded unless a clear connection to the European framework was established. Additionally, we limited the review to peer-reviewed academic articles to ensure the reliability of the sources. Articles not published in English were also excluded, as English is the predominant language in the scientific literature on this subject. In this way, using English ensures that researchers can understand and access the information.

### Search procedure and data collection

Searches were conducted in high-impact scientific databases, specifically Scopus and Web of Science. These databases were selected due to their extensive coverage and comprehensive indexing of topics related to industrial relations, labour policies, and European governance. The search strategy aimed to retrieve all possible results on ESD by applying Boolean operators in the title, abstract, or keywords, using the following query: ("European Social Dialogue" OR "European social dialogue" OR ("social dialogue" AND Europe)) ^1^. During this process, the acronym 'ESD' was excluded as it overlaps with the medical procedure 'Endoscopic Submucosal Dissection,' which significantly inflated the number of results without contributing relevant articles for analysis. Data collection was conducted on June 5, 2024, including all articles available in both databases up to that date.

Subsequently, using Covidence, a reviewer ^2^ carried out three rounds of screening of the studies until the sample to be extracted was concluded. After each of these rounds, the sample was reviewed by two additional researchers, finally including those records that had the entire team's agreement, proceeding in this way until the final extraction.

In total, 336 initial studies were retrieved and distributed as follows: 212 studies from Scopus and 124 from Web of Science. The first step in the process was the elimination of duplicate studies. A total of 110 duplicate references were identified and removed. The second screening phase evaluated the titles and abstracts of 226 studies selected after removing duplicates. During this phase, 113 studies that did not meet the inclusion criteria were excluded. These studies were discarded because they dealt with tangential issues, lacked empirical or methodological relevance to ESD, or were outdated regarding current EU policies. In the last phase, the full text of the remaining 113 studies was reviewed to assess their eligibility according to the predefined criteria. In this phase, 79 studies were excluded for the following reasons: 36 studies dealt with topics unrelated to the ESD (cross-industrial), 30 did not comply with the required academic format, and 13 were published in languages other than English. Thus, the final number of articles extracted for the review was 34.

**Figure 1.**
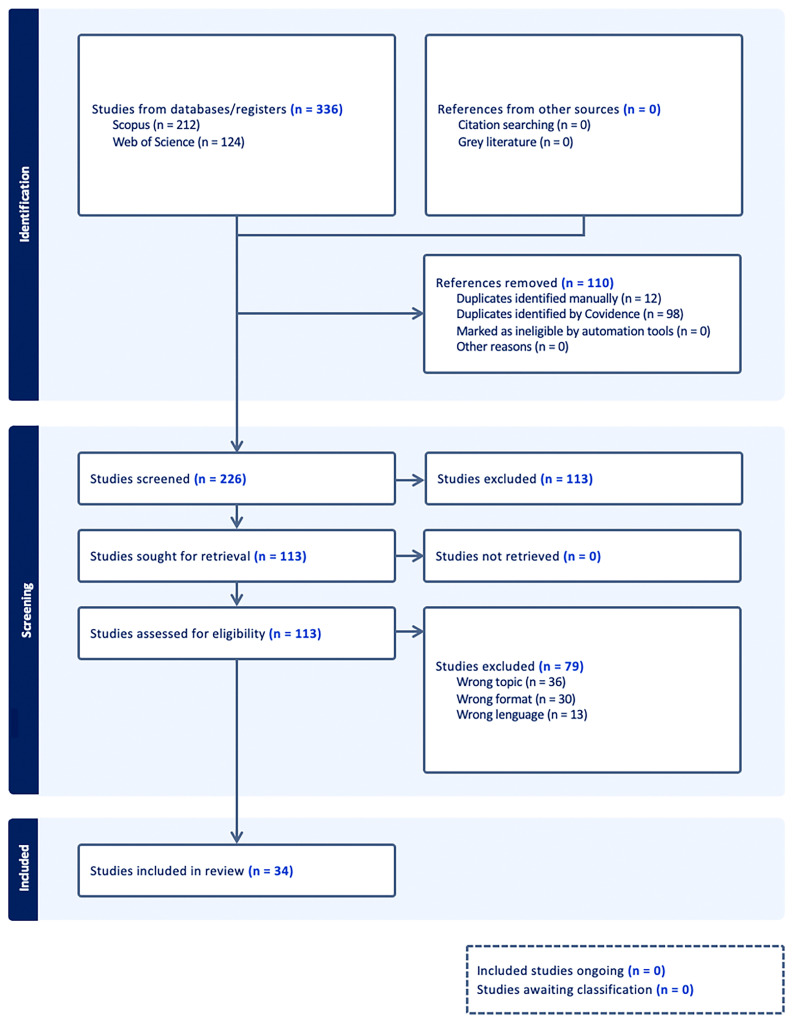
Figure 1 shows the information on the selection process following the PRISMA 2020 flow diagram.

Figure 1 shows the information on the selection process following the PRISMA 2020 flow diagram:

### Limitations of the study

The first limitation of this article relies on the fact that by including only studies published in English, relevant work in other languages may have been excluded. Also, by analysing only academic articles and excluding books and book chapters, our study may have overlooked complementary works on the subject. In addition, the fact that the acronym 'ESD' was removed from the query makes it possible to exclude some studies that deal slightly with the topic. Furthermore, the data in the articles analysed are understood to be reliable, as they have undergone peer review by academic journals. No sensitivity analyses were planned or performed, as this was a qualitative synthesis without aggregation models or weightings and without pre-specified alternative thresholds whose variation would provide additional evidence. Finally, although advanced search and reference management tools were used, there is always a risk of bias in the selection of studies, especially regarding the interpretation of their relevance and quality.

## 3. Results

Initially, a descriptive analysis of the information in the selected articles was conducted by examining the publication years, followed by an analysis of the journals. Finally, the keywords of the selected articles were analysed and grouped by subject.

**Figure 2.**
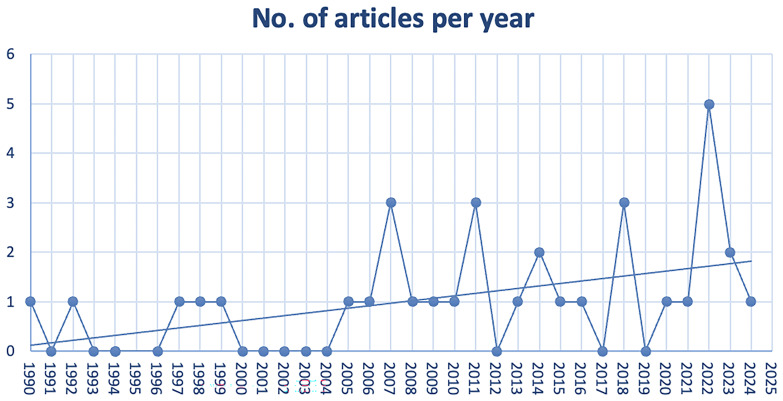
Figure 2 shows that studies on the ESD were published between 1990 and 2024, with a slight upward trend.

Figure 2 shows that studies on the ESD were published between 1990 and 2024, with a slight upward trend. This trend is likely due to two factors: the phenomenon's emergence and the tool's consolidation. Social dialogue began at the national level much earlier, but only took shape at the European level in the 1980s, materialising in 1992. Consequently, there was little literature before this period. The upward trend reflects the tool's consolidation, allowing scholars to analyse its impacts and failures through case studies.

Table 1

**Table 1.** Journals in which the articles analysed have been published. Own elaboration shows the journals where the analysed articles were published. There is a significant heterogeneity of journals covering topics ranging from labour relations and law to management and gender studies.

Journal	Area ^3^	No. of appearances
**European Journal of Industrial Relations**	Industrial Relations, Labour, and Employment	6
**Transfer: European Review of Labour and Research**	Industrial Relations, Labour, and Employment	3
**Common Market Law Review**	Law and Legal Studies	2
**International Labour Review**	Industrial Relations, Labour, and Employment	2
**Journal of European Social Policy**	Public Policy, Governance, and Politics	2
**Journal of Public Policy**	Public Policy, Governance, and Politics	2
**The International Journal of Human Resource Management**	Business and Management Studies	2
**West European Politics**	Public Policy, Governance, and Politics	2
**British Journal of Industrial Relations**	Industrial Relations, Labour, and Employment	1
**Business History**	Business and Management Studies	1
**Employee Relations**	Industrial Relations, Labour, and Employment	1
**European Constitutional Law Review**	Law and Legal Studies	1
**European Law Journal**	Law and Legal Studies	1
**Herald of the Russian Academy of Sciences**	Other	1
**Indiana Journal of Global Legal Studies**	Law and Legal Studies	1
**Industrial Law Journal**	Industrial Relations, Labour, and Employment	1
**International Journal of Environmental Research and Public Health**	Other	1
**Journal of East European Management Studies**	Business and Management Studies	1
**Journal of Education and Work**	Industrial Relations, Labour, and Employment	1
**Perspectives on Federalism**	Law and Legal Studies	1
**Women's Studies International Forum**	Other	1

Regarding the keywords, they were extracted and grouped into various categories and subcategories. For those articles without keywords, the Consensus version of GPT-4 was used to generate keywords based on an analysis of the body of each article. Subsequently, the same AI was asked to generate a classification of all available keywords, with categories and subcategories, to group them effectively. The result of this process is shown in Table 2.

**Table 2.** Categories and subcategories group keywords. Own elaboration.

Category ^4^	Frequency	%	Subcategory ^3^	Frequency	%
Employment, Labour, and Industrial Relations	90	51,43%	Dialogue Mechanisms and Partners	38	21,71%
			Education and Gender Issues	6	3,43%
			Health, Safety, and Employment Strategies	12	6,86%
			Labour Agreements and Bargaining	11	6,29%
			Labour Relations and Social Partners	17	9,71%
			Social Policies	6	3,43%
European Union Law and Governance	48	27,43%	EU Institutions, Regulations, and Bodies	15	8,57%
			EU Integration and Processes	14	8,00%
			EU Regulations and Policies	3	1,71%
			Lobbying and Representation	3	1,71%
			Treaties and Legal Frameworks	8	4,57%
			Types of Law	5	2,86%
Governance and Institutional Theories	22	12,57%	Governance Strategies and Theories	11	6,29%
			Institutional Change and Models	7	4,00%
			Risk and Management	4	2,29%
Political Economy and Policy	15	8,57%	Decision-Making and Voting	4	2,29%
			Economic Sectors and Policies	8	4,57%
			Theories and Models	3	1,71%

More than half of the keywords belong to industrial relations, so it can be determined that this is the predominant area of knowledge in this subject. European law and governance then occupy just over a quarter of the total keywords. Subsequently, the results include theoretical governance, institutional relations, and policymaking elements.

### Definition

The existing literature does not define ESD, generally resorting to the concept coined by international organisations. So, the definition of social dialogue provided by the International Labour Organisation (ILO) is used by Bisson (2022); Houtman *et al.* (2020). For the ILO, social dialogue refers to all types of negotiation, consultation or exchange of information between or among governments, employers and workers, whether bipartite or tripartite, and informal or institutionalised.

In turn, Akgüç *et al.* (2024) refer to the European Commission's definition of ESD, which includes negotiation, consultation, and information exchange among social partners and public authorities. ESD is a method to promote interaction on social and labour issues within the European framework. It prioritises consultation, negotiations, and agreements with social partners over legislative enactments proposed by the Commission and adopted by the Council (EUCO) without involving social partners.

Other authors seem to go beyond and, in contrast to the broader view of the ILO and the Commission, assert that European social partners themselves limit social dialogue to a bilateral interaction between workers and employers, even when it takes place under the framework of the Commission's consultations and according to the procedures laid down in the TFEU. According to Smismans (2008b), ESD is a procedure that allows social partners to address social issues through collective agreements rather than legislation. This means that the social partners can take direct control of the regulation of specific social and labour problems without the intervention of European public authorities. In this way, this definition emphasises the delimitation of the power of the public authorities and makes the social partners more autonomous.

This disparity of definitions makes some authors ( Prosser, 2016; Prosser & Perin, 2015; Sorensen *et al.*, 2022) highlight ESD's flexibility and adaptability to different contexts within the European Union. However, this also has certain limits. ESD does not cover all aspects of industrial relations. Strikes and lockouts were explicitly excluded from the legal scope of the dialogue, which limits its ability to address direct industrial disputes ( Prosser, 2016). Moreover, Social dialogue has a limited capacity to address issues of bogus self-employment, which may misclassify workers under this denomination in order to avoid employment benefits. EU regulations primarily handle these concerns through national laws rather than collective agreements or ESD ( Bandasz, 2014). Ultimately, the ESD cannot harmonise national social security systems. Although European legislation coordinates the social security systems of the Member States, the specific control of these systems is the responsibility of national legislation ( Bandasz, 2014; García-Muñoz-Alhambra, 2022; Pinto-Ramos, 2018).

### Regulation

ESD began to develop after the Treaty of Rome in 1957, though it was not a key element in early European integration. While the Treaty recognised the importance of improving labour conditions, its primary focus was on creating a common market ( Iankova, 2007). It was not until the 1980s that social dialogue started to institutionalise, notably under Jacques Delors, the European Commission President in 1985, who recognised the need to strengthen the social dimension of the European project to ensure the single market did not overshadow labour rights ( García-Muñoz-Alhambra, 2022).

A turning point was the Val Duchesse meetings in 1985, formally launching a dialogue between European trade unions and employers. This process continued with the Single European Act (SEA) of 1986, which integrated social dialogue into the acquis communautaire under Article 118b, obligating the Commission to promote dialogue between labour and management at the European level ( De Boer *et al.*, 2005). The Maastricht Treaty of 1992 formalised the role of social partners in shaping labour policy through the Social Policy Protocol, and the Amsterdam Treaty of 1997 further consolidated these roles in the core EU treaties ( Keller & Sörries, 1999; Keune & Marginson, 2013).

Through Articles 154 and 155 of the TFEU ( Begega & Aranea, 2018; Bisson, 2022; Guardiancich *et al.*, 2023), social partners were granted the ability to negotiate framework agreements that could be transformed into EU directives or implemented autonomously under national labour laws ( Keune & Marginson, 2013). Unlike other EU legislative processes, the European Parliament has no formal role in negotiations under these articles, and the Member States ultimately decide on agreements in the EUCO, which can lead to potential political challenges in implementation ( Bandasz, 2014; García-Muñoz-Alhambra, 2022).

Moreover, these articles enable social partners to share legislative initiative power with the Commission on labour and social policies ( Carré & Steiert, 2022), through the compulsory consultation with social partners. So, Article 154 TFEU requires the European Commission to consult social partners before legislative proposals (Article 154 TFEU). In turn, Article 155 TFEU allows social partners to negotiate agreements that the EUCO may adopt into binding law ( Carré & Steiert, 2022; McDougall, 2022).

Initially, agreements like those on parental leave and working time were often implemented as binding directives. However, there has been a gradual shift towards soft law since the 2000s, with social partners increasingly adopting autonomous agreements implemented voluntarily by Member States or via national labour practices ( Prosser, 2011). This trend towards soft law is part of a broader governance shift in Europe, exemplified by the Open Method of Coordination (OMC) introduced by the Amsterdam Treaty. The OMC allows for non-binding guidelines on employment, offering an alternative to binding legislation while maintaining the sovereignty of Member States over social policy ( Pinto-Ramos, 2018). This trend was consolidated at the Laeken Summit (2001), where the social partners advocated autonomous and bipartite social dialogue ( Bisson, 2022; De Boer *et al.*, 2005; González-Begega & Aranea, 2018) aimed at voluntary and non-legally binding agreements ( De Boer *et al.*, 2005; Prosser, 2016; Sorensen *et al.*, 2022). Although this approach provides flexibility, it has raised concerns over its effectiveness in harmonising labour standards across the EU ( Pinto-Ramos, 2018).

### Actors

There are two types of actors involved in Social Dialogue, according to the TFEU. On the one hand, there are the 'public authorities', i.e. the EUCO and the European Commission. On the other hand, the 'social partners' must be considered. Although the term' social partner' was not explicitly defined in the analysed articles, the criteria for their inclusion in the ESD are detailed in the literature. To be considered as a social partner at the European level, organisations must: (1) operate across multiple industries or be relevant to specific sectors or categories and be structured at the European level; (2) be integral and recognised components of national social partner frameworks, with the ability to negotiate agreements, and should aim to be representative of all Member States; (3) have sufficient structures in place to ensure effective participation in the consultation process ( Franseen & Jacobs, 1998; Keller & Sörries, 1999; Verbruggen, 2009).

Thus, three participants in the Val Duchesse Summit were initially invited to the ESD as social partners. These are the : European Trade Union Confederation (ETUC), Union des Industries de la Communauté européenne (UNICE)/BusinessEurope and the Centre Européen des Entreprises à Participation Publique et des Entreprises d'Intérêt Economique Général (CEEP)/Services of General Interest Europe (SGI Europe) ( Cockburn, 1997; De Boer *et al.*, 2005; Ertel *et al.*, 2010; Franseen & Jacobs, 1998).

ETUC is an umbrella organisation for workers' trade unions in various European countries. It was founded in 1973 to represent and protect workers' interests at the European level, mainly in the institutions of the European Union, such as the European Commission, the European Parliament and the EUCO ( Adamczyk, 2018; Begega & Aranea, 2018). One of ETUC's key objectives is to influence European policies in areas such as employment, social protection, working conditions and vocational training ( Erne, 2008).

BusinessEurope is the leading organisation representing businesses and employers in Europe. Founded in 1958 as UNICE (Union of Industrial and Employers' Confederations of Europe), it changed its denomination in 2007 to its current name ( Guardiancich *et al.*, 2023). This organisation acts as the voice of business at the European level, defending its interests before the leading institutions of the European Union, such as the European Commission, the European Parliament and EUCO ( Guardiancich *et al.*, 2023). This organisation defines its primary objective as promoting a favourable European business environment that fosters competitiveness, economic growth, and job creation.

SGI Europe, formerly known as CEEP, ‘Centre Européen des Entreprises à Participation Publique et des Entreprises d'Intérêt Economique Général’, is an organisation representing companies and organisations that manage services of general interest in Europe. Services of general interest (SGI) refer to economic activities that meet fundamental public needs and are often provided by public and private entities under state regulation or through specific concessions ( Keller & Sörries, 1999). SGI Europe brings together both public and private companies operating in these sectors, acting as an interlocutor in the ESD, especially on issues related to the provision of public services and their regulation ( Franseen & Jacobs, 1998).

Upon identifying the key participants in the social dialogue, the literature highlights the essentiality of examining the roles of additional stakeholders whose presence or absence may warrant further consideration. On the side of the public authorities, it is essential to note that the European Parliament has no role in the ESD. Thus, the only European institution whose members are directly elected by European citizens is excluded from this negotiation process ( Franseen & Jacobs, 1998; García-Muñoz-Alhambra, 2022; Guery, 1992; Keller & Sörries, 1999).

Social partners, workers, employers, and public companies are represented. However, small and medium-sized enterprises were not initially recognised as social partners. This type of business tends to set up different employers' organisations from large corporations, as their interests differ. Small and medium-sized enterprises are represented by SMEUnited, previously known as the 'Union Européenne de l'Artisanat et des Petites et Moyennes Entreprises' (UEAPME). Founded in 1980, SMEUnited was already active in social dialogue at the time of the Val Duchesse meetings. However, it was not formally recognised as a complete social partner until the Maastricht Social Protocol and the Amsterdam Treaty were revised ( Guardiancich *et al.*, 2023). This formal recognition, granted in 1999, enabled UEAPME, now SMEUnited, to gain greater negotiation autonomy and be officially included in the autonomous social dialogue mechanisms. This recognition came after the UEAPME case. UEAPME sued the European Commission for excluding it from social agreement negotiations, arguing that it lacked the same legal status as other organisations, such as BusinessEurope and ETUC. The Court of Justice of the European Union ruled in favour of UEAPME, allowing its inclusion in the social dialogue process on equal footing with other social partners ( Franseen & Jacobs, 1998).

With this set of actors, ESD becomes a figure of co-regulation ( Keune & Marginson, 2013; Schömann, 2011), understood as a regulatory approach that involves collaboration between private and public entities to govern specific interests and achieve objectives ( Verbruggen, 2009). In this way, the ESD is a tool for private institutions (that defend sectors of the population) to influence policymaking on the issues that affect them, instead of the classical representation by political parties ( Velluti, 2022).

### Relations

***The process of bargaining***

The relations between social partners and European bodies are many and varied. There are two primary forms within the ESD: consultations by the Commission and autonomous negotiations by the social partners.

Following the Maastricht Treaty (1992), the European Commission must consult the social partners in areas related to employment, working conditions, social security and other social issues ( Keller & Sörries, 1999; Prosser, 2011). This process is divided into two phases. In the first phase, the European Commission consults the social partners to determine whether they consider that action at the European level is necessary. If the social partners agree on the need to address an issue, they may choose to enter into the second phase, having direct negotiations with each other, with up to nine months to reach an agreement. During this period, the Commission refrains from further action ( Keller & Sörries, 1999; Léonard, 2008). The EU institutions will only intervene if the social partners have not reached an agreement within the specified period or if the organisations decide not to negotiate ( Carré & Steiert, 2022). In turn, the social partners at the European level can negotiate autonomously; for their agreements to be binding, they must address issues within the EU's competence and receive approval from the EUCO ( McDougall, 2022).

When the social partners reach an agreement, it can be implemented in two ways. First, it can be implemented autonomously at the national level through the mechanisms established by the social partners in each Member State. Alternatively, if a broader impact is desired, the social partners can request the Commission to submit the agreement to the EUCO for implementation as a binding directive or regulation ( De Boer *et al.*, 2005; Keller & Sörries, 1999; Schömann, 2011). Thus, the Commission must ask the EUCO to approve a binding agreement between the social partners. Moreover, in this process, the Commission has absolute power to approve or not the agreement reached by the social partners. This process raises doubts about the independence of the social partners to negotiate ( Prosser, 2016), as they need the Commission's support to turn their agreements into binding legal texts ( García-Muñoz-Alhambra, 2022; Verbruggen, 2009).

***Decision-making process determinants***

Four key points linked to the decision-making process should be highlighted. The first is that the social partners' expectations of benefit condition their willingness to participate or not. Thus, for an issue to be discussed and negotiated within the ESD process, all social partners must participate, and they will only do so if they can achieve certain benefits for those they represent ( Ertel *et al.*, 2010; Houtman *et al.*, 2020; Prosser & Perin, 2015). However, this willingness may be conditioned by the Commission, since, due to its position, it has tools of pressure, being able to make the social partners negotiate ( Ertel *et al.*, 2010).

The second point relies on the interests of the social agents. As expected, the different social partners have divergent interests, which lead them to diverse dynamics and strategies. For instance, European employers generally favour free markets over regulated ones, showing a structural resistance to binding institutions ( Ertel *et al.*, 2010). This often makes them adopt resistance tactics or advocate for maximum subsidiarity when they cannot block such regulations ( Guardiancich *et al.*, 2023; Smismans, 2008b; Spyropoulos, 1990).

Moreover, regarding the barriers workers and their unions face, Smismans (2008b) notes that workers lack the bargaining power to pressure employers to engage in negotiations on key social issues. This is exacerbated by the lack of homogeneity between unions in different member states, whose priorities and interests may differ considerably. This trade union fragmentation weakens the capacity for collective action and hinders the formation of a coherent European-level labour agenda ( Sorensen *et al.*, 2022).

Finally, we mention the influence of the European Commission on the ESD, which has often been conceptualised as the 'shadow of the hierarchy' (SoH). This concept, key to understanding the dynamics of social partners' negotiations at the European level, describes how the Commission's explicit or implicit threat of legislative action incentivises employers and trade unions to participate actively in negotiating agreements ( Ertel *et al.*, 2010). Legislative pressure is thus indispensable to stimulate and maintain the commitment of the social partners, especially in reaching binding agreements, since, in the absence of incentives provided by the European institutions, the social partners are unlikely to reach a consensus ( Keller & Sörries, 1999; Smismans, 2008a). However, although SoH is a necessary condition for binding agreements, it is insufficient to guarantee their success ( Sorensen *et al.*, 2022).

### Structures

ESD is not a linear or monolithic process. It offers social actors a variety of pathways to achieve their objectives. One critical dimension of the ESD is the level of action, which is divided into two primary frameworks: the European Cross-sectoral Social Dialogue (ECISD) and the European Sectoral Social Dialogue (ESSD). The ECISD operates at a broad, overarching level, while the ESSD focuses on specific productive sectors ( González-Begega & Aranea, 2018; Keller & Sörries, 1999; McDougall, 2022).

**Figure 3.**
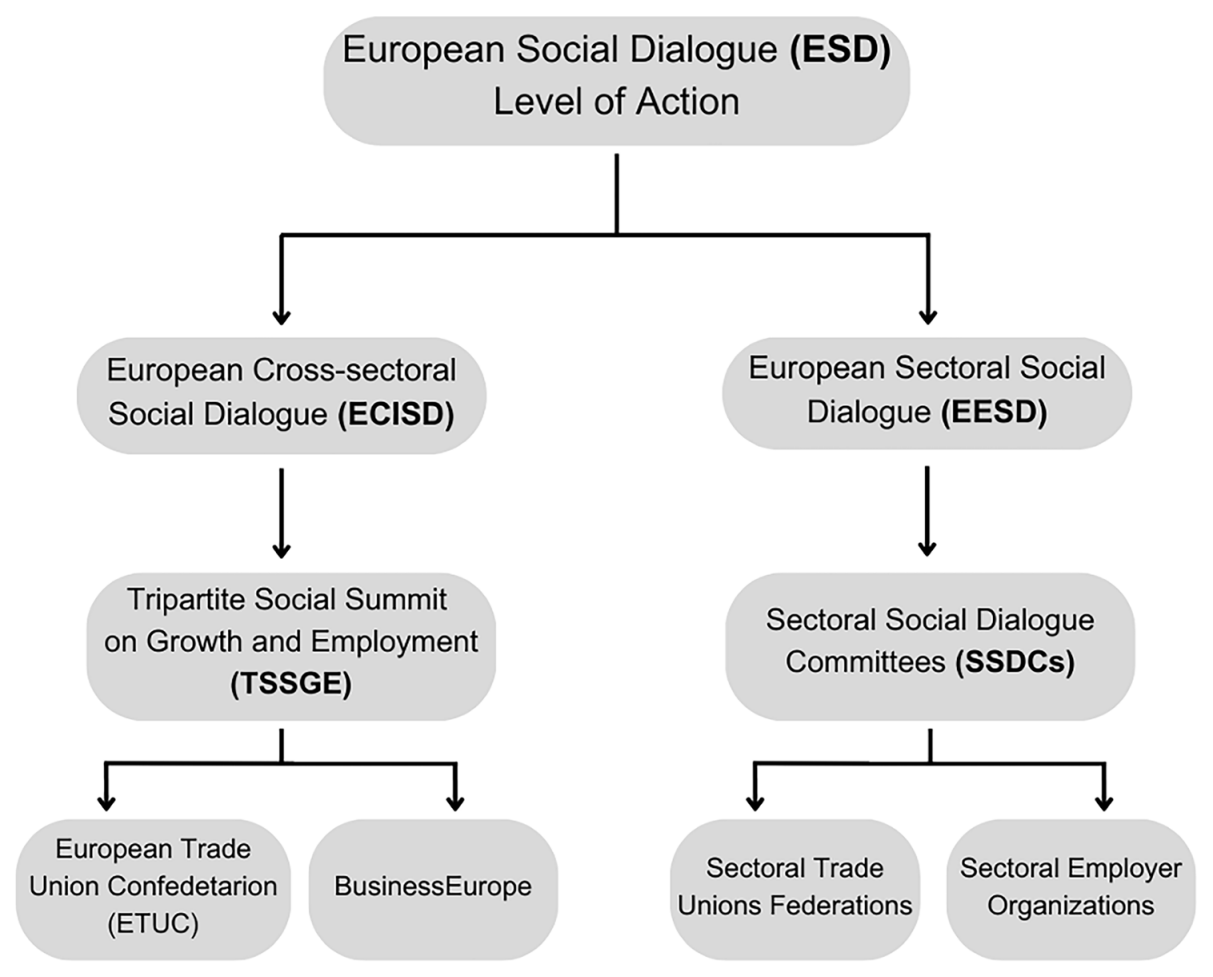
Figure 3 shows the different structures of the ESD and the actors involved.

This differentiation results in distinct structures governed by the same principles. At the cross-sectoral level, the Tripartite Social Summit on Growth and Employment (TSSGE)—formerly the Tripartite Social Summit (TSS)—serves as the primary platform for high-level cross-industry negotiations and is formed by the employers' organisations, the unions and the EU institutions ( González-Begega & Aranea, 2018). In contrast, the Sectoral Social Dialogue Committees (SSDCs) provide a bipartite framework for sector-specific negotiations, with 43 committees facilitating dialogue between European social partners and are formed by the sectoral sections of the employers' organisations and the trade unions ( González-Begega & Aranea, 2018; Sorensen & Dumay, 2023). Accordingly, European-level social dialogue operates on two interrelated levels: the pan-European cross-sectoral level, where organisations such as the European Trade Union Confederation (ETUC) and BusinessEurope conduct dialogue, and the sectoral level, where sectoral federations and employer representatives focus on economic sectors ( McDougall, 2022). For a better comprehension, Figure 3 shows the different structures of the ESD and the actors involved.

Despite this structural division, ambiguities persist regarding the competencies of each body. While the TFEU defines the general scope of ESD in Article 153, it does not explicitly differentiate the competencies of the ECISD and the ESSD. Similarly, the academic literature lacks a clear consensus on the distinction between these two dialogue levels.

Further complicating the framework is the fact that ESD does not encompass all issues. Some matters, such as wage regulation, remain under national jurisdiction ( González-Begega & Aranea, 2018). Additionally, in certain countries, competencies are further devolved to sub-national levels, adding another layer of complexity. This intricate system reflects the broader concept of multi-level governance, consolidating horizontal and vertical relationships among public and private actors while fostering interdependence between various structures involved in the ESD process ( Bandasz, 2014; Bisson, 2022; Keller & Sörries, 1999; Keune & Marginson, 2013; McDougall, 2022).

### Outcomes

***Types of agreements***

In its various forms, ESD offers a wide range of possible outcomes. The literature highlights several examples, including agreements, binding and non-binding recommendations, declarations, collective agreements, social pacts, and bipartite documents such as joint opinions, frameworks for action, codes of conduct, guidelines, and handbooks ( Akgüç *et al.*, 2024; Smismans, 2008b). These outcomes can be grouped into two categories: binding and non-binding agreements ( González-Begega & Aranea, 2018; Sorensen & Dumay, 2023). Since the early 2000s, with the introduction of the OMC, there has been a clear trend favouring non-binding agreements over binding ones ( Schömann, 2011).

Two categories can be distinguished depending on the Commission's role in the process: guided or autonomous social dialogue. While in the guided ESD, the Commission initiates and encourages social partners to agree on specific issues ( Bisson, 2022), in the autonomous ESD, the social partners reach an agreement without the mediation of the EU public authorities. In both cases, either the agreement is promoted by the Commission or autonomously by the social partners, if they finally decide that its implementation will be autonomous, it is the social partners who must ensure compliance with the agreement, as there is no legal obligation to comply with such agreements ( Smismans, 2008b).

**Figure 4.**
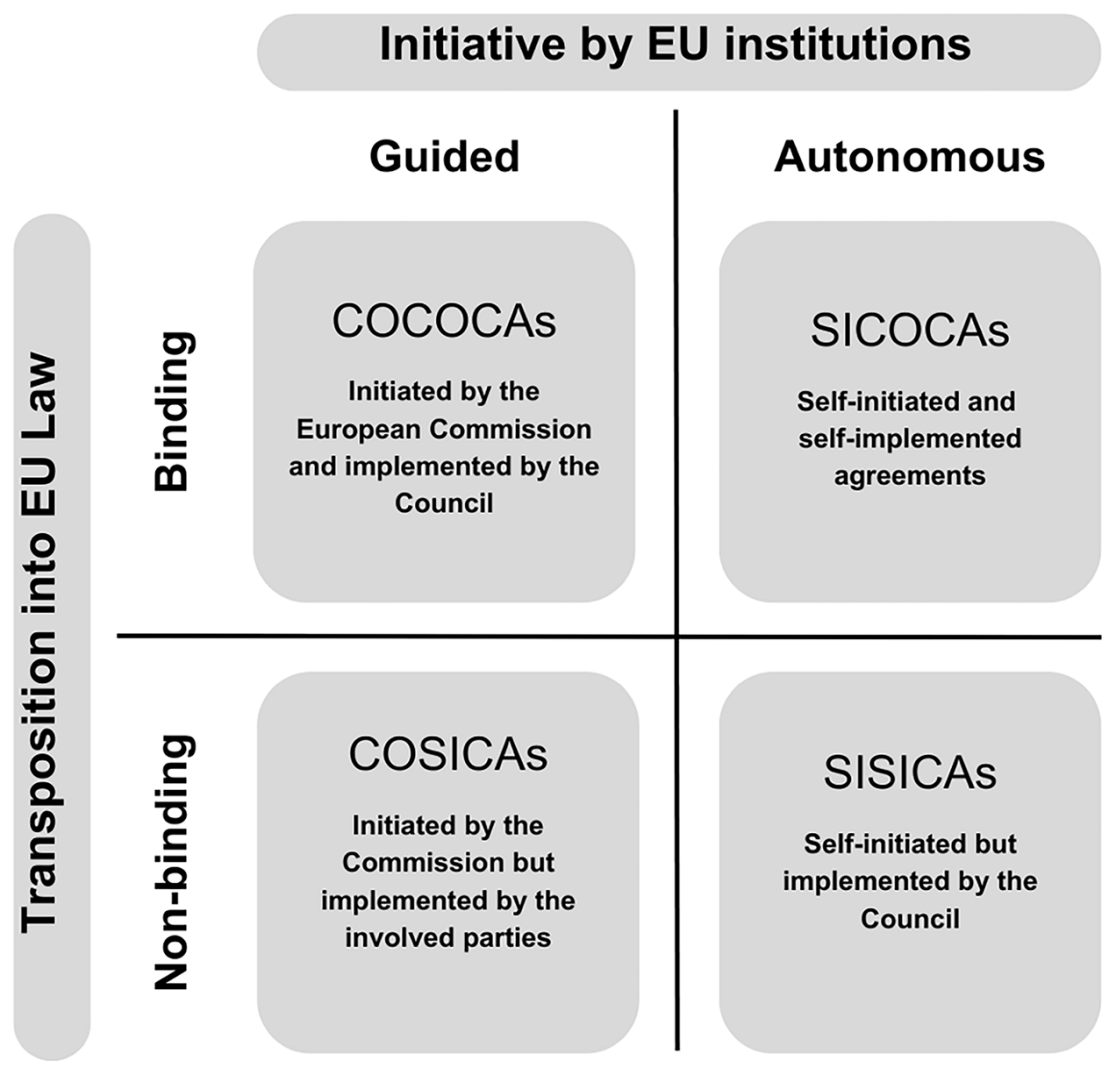
Figure 4 reflects those divisions of agreements.

Among the binding agreements, collective agreements are particularly significant. These agreements have a unique character in industrial relations due to their binding nature and coverage of many workers. Several types of collective agreements exist, differing in how they are initiated and implemented. COCOCAs are collective agreements that the European Commission initiates, and the EUCO implements. COSICAs are agreements initiated by the Commission but implemented directly by the involved parties. SISICAs refer to agreements that are both self-initiated and self-implemented by the social partners. Lastly, SICOCAs are self-initiated agreements implemented by the EUCO. These types reflect varying levels of involvement by the European Commission, the EUCO, and social partners in the initiation and implementation processes ( Smismans, 2008b). For a better comprehension, Figure 4 reflects those divisions of agreements.

***Effectiveness of the agreements***

Bandasz (2014) claims that less than 2% of all documents adopted at the sectoral level constitute agreements, and less than 10% are expected to have any impact at the national level, as most texts function as lobbying instruments addressed to the EU institutions. Similarly, several scholars attribute the limited effectiveness of the ESD to this scarcity of binding agreements ( Pinto-Ramos, 2018; Prosser, 2011; Verbruggen, 2009). Unfortunately, no public data on the number of agreements and their share of applications is available.

This weakness is further exacerbated by structural imbalances between trade unions and employers, driven by disparities in both means and objectives. Concerning means, the exclusion of the right to strike within the ESD undermines trade unions' ability to exert pressure ( Bandasz, 2014). Regarding objectives, the thematic fragmentation of the ESD relegates crucial industrial relations matters, such as wage bargaining, to national frameworks, thereby reducing its influence to that of a negotiation platform ( Prosser, 2011). Finally, other studies point to a lack of political will to act on social policy issues in the EU, which limits the ESD ( Schömann, 2011).

However, the ESD is revealed as an instrument relevant to the EU. Firstly, it helps harmonise European social regulation, making it possible to equalise Member States' legislation while considering social partners ( Bandasz, 2014; González-Begega & Aranea, 2018). Moreover, non-binding agreements between social partners can reach places where governmental action does not, offering soft governance that legislative production cannot get ( Smismans, 2008b).

## 4. Discussion

The systematic review conducted on the ESD provides a comprehensive understanding of its definition, regulatory framework, actors, relationships, structures, and outcomes. By synthesising insights from the literature, it is possible to identify the strengths and limitations of this key mechanism within the EU governance system.

The ESD has demonstrated a relevant capacity to influence labour policies and ensure the participation of social partners in decision-making processes at the EU level. However, the lack of a universally agreed-upon definition of ESD, as identified in the literature, remains a critical challenge. While the European Commission provides a broad definition, some authors argue that European social partners limit the scope of ESD to bipartite interactions, excluding public authorities from direct negotiation processes ( Bisson, 2022; Smismans, 2008b). This conceptual ambiguity may hinder the consistent application of ESD across member states and reduce its overall impact.

Regarding its regulation, the historical development of the ESD reflects its evolution from a consultative mechanism under the Treaty of Rome (1957) to a formalised governance tool under the Maastricht (1992) and Amsterdam Treaties (1997). The literature highlights the role of Articles 154 and 155 of the TFEU in empowering social partners to negotiate agreements that can become binding EU directives ( González-Begega & Aranea, 2018; Keller & Sörries, 1999). Nevertheless, the increasing reliance on non-binding agreements and soft law mechanisms since the early 2000s raises concerns about the declining legislative influence of ESD ( Pinto-Ramos, 2018; Prosser, 2011). This shift to non-binding support has led to questions regarding the enforcement capacity of ESD agreements, particularly in contexts where compliance relies solely on voluntary adoption by member states.

The actors involved in ESD reflect a structured but limited representation of social interests. The primary social partners, including ETUC, BusinessEurope, and SGI Europe, have established a stable presence within ESD processes ( Cockburn, 1997; De Boer *et al.*, 2005). However, the exclusion of other entities, such as SME United, until legal challenges forced their inclusion illustrates the ongoing struggles for broader representativeness ( Guardiancich *et al.*, 2023). Enhancing the inclusivity of actors by incorporating broader civil society organisations and minor sectoral interests could further legitimise and diversify the ESD process. Furthermore, the absence of the European Parliament from the negotiation process raises questions about democratic legitimacy ( Franseen & Jacobs, 1998). There are two reasons for this. The first is that the legitimacy of the public bodies involved is indirect, as the democratic nature of both the EUCO and the Commission is indirect. The second relates to the representativeness of the social partners.

According to Pitkin (2023), representation can be categorised into four distinct types. The formalist type emphasises institutional procedures and mechanisms that authorise and hold representatives accountable. The symbolic type explores how representatives embody values, identities, or aspirations recognised and accepted by the represented. The descriptive type focuses on the alignment between the representative's characteristics (such as gender, race, or class) and those of the represented, under the premise that shared traits enhance understanding and advocacy. Finally, the substantive type prioritises the representative's actions in advancing the interests of the represented, regardless of shared characteristics. Together, these types capture the intricate nature of political representation. According to this classification, the representativeness of government bodies is substantive but also formalistic and symbolic. However, social partners have a substantive representativeness, as do public institutions, but also a descriptive one. This fact turns the concept of representativeness upside down. The individual is not only represented as a citizen of one of the member states but also as a consequence of their role in the labour market. Thus, due to the ESD, new types of representativeness are added to the EU industrial relations system, therefore improving its representativeness. Similarly, Velluti (2022) observes that the creation of the ESD may signal a shift from the concept of representation to that of representativeness. While not a rejection of representation, this shift is described as a 'heteronomist alternative', prioritising the selection of trade union organisations capable of fulfilling new 'public functions' over strengthening robust associative relationships between the represented and their representatives. Despite the terminological distinctions, the conclusion remains consistent: the ESD enhances the connection between representatives and those they represent.

The relationships between actors within the ESD reveal complex dynamics. The negotiation process often depends on the European Commission's ability to influence social partners through the 'shadow of hierarchy' approach, where the threat of legislative action encourages cooperation ( Ertel *et al.*, 2010; Smismans, 2008a). However, the power imbalance between employers and trade unions, with the latter facing reduced bargaining power due to tricky negotiation mechanisms, further complicates these interactions ( Bandasz, 2014; Prosser, 2011). National-level variations in union strength can exacerbate this imbalance, further limiting some social partners' ability to shape policy outcomes effectively.

The structural aspects of ESD, divided into cross-sectoral and sectoral frameworks, provide flexibility for different policy areas. However, the literature reveals ambiguities regarding the distribution of competencies between the ECISD and the ESSD ( González-Begega & Aranea, 2018; McDougall, 2022). These structural ambiguities may contribute to inconsistent implementation and reduced policy coherence across sectors. Additionally, while beneficial in some contexts, the proliferation of sectoral committees raises concerns about fragmentation and the ability to coordinate policy strategies effectively across the EU, as it reduces the area of negotiation and excludes important issues that can only be dealt with at the cross-sectoral level.

While varied, ESD outcomes highlight a declining production of binding agreements, with non-binding outcomes becoming increasingly common since the introduction of the OMC ( Schömann, 2011). Although non-binding agreements can extend political influence to areas beyond legislative reach and even push for legislative change, they often lack enforceability, making their impact uncertain ( Bandasz, 2014; Pinto-Ramos, 2018). For example, the rise of framework agreements and voluntary guidelines has led to policy diffusion, but often without sufficient mechanisms for monitoring compliance or assessing long-term impact. Thus, despite their limited impact, the few regulatory changes aim to harmonise labour rights upward, improving working conditions for millions across Europe ( Bandasz, 2014; González-Begega & Aranea, 2018). Moreover, the ESD serves as an alternative law-making mechanism, enhancing the democratic legitimacy of European institutions by enabling social partners to actively shape public policy and advocate for the interests of those they represent ( Carré & Steiert, 2022).

### Proposals for ESD improvement

The review of the key aspects of the ESD leads us to propose practical measures to make it more effective and relevant to current challenges.

Firstly, promulgating a single directive on this tool is considered pertinent. This would improve its clarity and uniformity, providing a clear and harmonised framework for promoting and guaranteeing social dialogue in all Member States. In this way, the definition of social dialogue would be clarified, and its regulation would be more straightforward, increasing its effectiveness. Moreover, such a development would reflect a consolidated EU commitment to ensure that the social partners have a role in economic and social governance, strengthening the EU industrial relations system.

Secondly, the issue of non-compliance with autonomous agreements has been brought to attention. While it is recognised that public authorities cannot intervene in these types of agreements, it is suggested that the Social Dialogue Committee, along with the sectoral committees, regularly prepare reports on the status of these agreements and make them publicly accessible. By doing so, this information can help enhance adherence to the agreements, with both the social partners and civil society serving as a control mechanism.

Finally, the creation of a European Social Dialogue Transparency and Evaluation Board (ESDTEB) is proposed. Linked to the Social Dialogue Committee, its purpose would be to collect, analyse, and disseminate data on the development and impact of this mechanism within the European Union. Its structure could consist of a governing council made up of representatives from the European Commission, social partners, and independent experts, along with departments specialised in data analysis and communication. The ESDTEB would be responsible for producing and assessing key indicators such as the number of agreements reached, the level of sectoral participation, the reduction of labour disputes, and the degree of implementation by Member States. Through an interactive digital portal, the organisation would publish accessible statistics and analyses, contributing to the continuous improvement of social dialogue. This initiative aims to strengthen social dialogue as a more transparent, inclusive, and effective tool, supported by data that demonstrate its impact and help to optimise its outcomes.

## 5. Conclusions

This literature review has revealed the blurry nature of ESD, a tool with high potential but still numerous deficiencies. It has been highlighted that while ESD remains a vital mechanism for participatory governance in the EU, its effectiveness is constrained by conceptual ambiguities, limited representation, power imbalances, and a shift towards non-binding agreements.

Addressing these challenges requires a more evident definitional consensus, enhanced inclusivity among social partners, and a balanced regulatory approach to ensure both flexibility and enforceability. Additionally, leveraging digital tools for improved transparency and expanding the ESD's focus to address emerging labour market challenges, such as platform work and the gig economy, could further strengthen its relevance. The findings of this systematic review offer a critical foundation for further research on improving the operational efficiency and democratic legitimacy of ESD, helping to bring clarity to a necessary debate that will shape the future of industrial relations in the EU.

As the EU faces emerging challenges and increasing labour market fragmentation, ESD must evolve to address these contemporary issues. Expanding its scope to include these areas will ensure its continued relevance as a governance mechanism. While ESD has proven its value as an alternative form of democratic law-making, addressing its conceptual and operational limitations is essential to maximise its impact on European labour relations. To achieve this, some changes need to be made to the ESD to facilitate its operation, enhance its strengths, reduce its weaknesses, and encourage the various actors to use this collective bargaining tool.

This systematic review lays the basis for future research and policy innovations that strengthen the role of the ESD in shaping a socially cohesive and economically resilient Europe, unifying the labour systems of the different member states and ensuring decent working conditions and business benefits.

## Ethics & consent

Ethical approval and consent were not required for this study.

## Data Availability

The data supporting the findings of this study are openly available in the Open Science Framework (OSF) repository under the title *Revisiting European Social Dialogue: A Systematic Literature Review*. The dataset has been assigned the following DOI: 10.17605/OSF.IO/3HYGU ( https://osf.io/3hygu/). Cárdenas-Domínguez, F., García, M. F., & Gerbeau, Y. M. (2025, September 24). Revisiting European Social Dialogue: A Systematic Literature Review. https://doi.org/10.17605/OSF.IO/3HYGU. Here you can also find the protocol and other elements related to the PRISMA 2020 statement. The materials are distributed under the CC-BY Attribution 4.0 International license.

## References

[ref-1] Adamczyk S : Inside the trade union family: the 'two worlds' within the European Trade Union Confederation. *Eur J Ind Relat.* 2018;24(2):179–192. 10.1177/0959680118760630

[ref-2] Akgüç M Kahancová M Masso J : One-way street to the European Union? Between national and EU-level social dialogue 20 years after eastward EU enlargement. *Transfer Eur Rev Labour Res.* 2024;30(1):33–49. 10.1177/10242589241229070

[ref-3] Bandasz K : A framework agreement in the hairdressing sector: the European social dialogue at a crossroads. *Transfer Eur Rev Labour Res.* 2014;20(4):505–520. 10.1177/1024258914538914

[ref-4] Begega SG Aranea M : The establishment of a European industrial relations system: still under construction or chasing a chimaera?In: *Employee Relations.*Emerald Group Holdings Ltd,2018;40(4):600–616. 10.1108/ER-07-2017-0151

[ref-5] Bisson LS : European Social Dialogue: history, characteristics, and perspectives. *Her Russ Acad Sci.* 2022;92(S7):S660–S666. 10.1134/S1019331622130147

[ref-6] Carré P Steiert M : Social Europe without social dialogue: decision of the Court of Justice of the European Union in C-928/19 P *European Federation of Public Service Unions*. *Eur Const Law Rev.* 2022;18(2):315–333. 10.1017/S1574019622000177

[ref-7] Cockburn C : Gender in an international space: trade union women as European social actors. *Womens Stud Int Forum.* 1997;20(4):459–470. 10.1016/S0277-5395(97)00035-6

[ref-8] De Boer R Benedictus H Van Der Meer M : Broadening without intensification: the added value of the European social and sectoral dialogue. *Eur J Ind Relat.* 2005;11(1):51–70. 10.1177/0959680105050400

[ref-9] Erne R : European Unions: labour's quest for a transnational democracy.ILR Press; Cornell University Press,2008. Reference Source

[ref-10] Ertel M Stilijanow U Iavicoli S : European social dialogue on psychosocial risks at work: benefits and challenges. *Eur J Ind Relat.* 2010;16(2):169–183. 10.1177/0959680110364830

[ref-11] Franseen E Jacob ATJM : The question of representativity in the European social dialogue. *Common Market Law Rev.* 1998;35(6):1295–1312. 10.54648/199393

[ref-12] García-Muñoz-Alhambra MA : An uncertain future for EU-Level collective bargaining: the new rules of the game after *EPSU*. *Ind Law J.* 2022;51(2):318–345. 10.1093/indlaw/dwac006

[ref-39] Gómez Urquijo LT : El diálogo social en la definición de la estrategia económica y social de la Unión Europea. *Oñati Socio-Legal Series.* 2024;14(S7). Reference Source

[ref-13] González-Begega S Aranea M : The establishing of a European industrial relations system: still under construction or chasing a chimera? *Employee Relations.* 2018;40(4):600–616. 10.1108/ER-07-2017-0151

[ref-14] Guardiancich I Terlizzi A Natali D : The social policy preferences of EU employers' organisations: an exploratory analysis. *Eur J Ind Relat.* 2023;29(3):243–269. 10.1177/09596801231153928

[ref-15] Guery G : European collective bargaining and the Maastricht Treaty. *Int Labour Rev.* 1992;131(6):581–599.

[ref-16] Houtman I van Zwieten M Leka S : Social dialogue and psychosocial risk management: added value of manager and employee representative agreement in risk perception and awareness. *Int J Environ Res Public Health.* 2020;17(10):3672. 10.3390/ijerph17103672 32456084 PMC7277720

[ref-17] Iankova EA : Europeanization of social partnership in EU-acceding countries. *J East Eur Manag Stud.* 2007;12(4):297–317. 10.5771/0949-6181-2007-4-297

[ref-18] Im Z Larsen TP Pircher B : European Social Dialogues: shaping EU social policy through parental leave rights. *ILR Rev.* 2024;77(5). 10.1177/00197939241231789

[ref-19] Keller B Sörries B : The new European social dialogue: old wine in new bottles? *J Eur Soc Policy.* 1999;9(2):111–125. 10.1177/095892879900900202

[ref-20] Keune M Marginson P : Transnational industrial relations as multi-level governance: interdependencies in European Social Dialogue. *Br J Ind Relat.* 2013;51(3):473–497. 10.1111/bjir.12005

[ref-21] Léonard E : European Sectoral Social Dialogue: an analytical framework. *Eur J Ind Relat.* 2008;14(4):401–419. 10.1177/0959680108097493

[ref-29] Martín RN Visser J : A more "Autonomous" European Social Dialogue: the implementation of the framework agreement on telework. *Int J Comp Labour Law Ind Relat.* 2008;24(4):511–548. 10.54648/ijcl2008027

[ref-22] McDougall P : European cross-sectoral collective bargaining as post-crisis social policy. *Indiana J Glob Legal Stud.* 2022;29(1):163–215. Reference Source

[ref-23] Page MJ McKenzie JE Bossuyt PM : The PRISMA 2020 statement: an updated guideline for reporting systematic reviews. *BMJ.* 2021;372:n71. 10.1136/bmj.n71 33782057 PMC8005924

[ref-24] Pinto-Ramos M : Reconstructing social dialogue. *Perspectives on Federalism.* 2018;10(1):147–175. 10.2478/pof-2018-0008

[ref-25] Pitkin HF : The concept of representation.University of California Press,2023.

[ref-26] Prosser T : The implementation of the telework and work-related stress agreements: European social dialogue through "soft" law? *European Journal of Industrial Relations.* 2011;17(3):245–260. 10.1177/0959680111410964

[ref-27] Prosser T : Economic union without social union: the strange case of the European social dialogue. *J Eur Soc Policy.* 2016;26(5):460–472. 10.1177/0958928716664298

[ref-28] Prosser T Perin E : European tripartism: chimera or reality? The 'new phase' of the European social dialogue in the light of tripartite theory and practice. *Business History.* 2015;57(3):376–397. 10.1080/00076791.2014.983481

[ref-30] Schömann I : EU integration and EU initiatives on employee participation and social dialogue. *Transfer Eur Rev Labour Res.* 2011;17(2):239–249. 10.1177/1024258911401526

[ref-31] Smismans S : The European Social Dialogue in the shadow of hierarchy. *J Public Policy.* 2008a;28(1):161–180. 10.1017/S0143814X08000822

[ref-32] Smismans S : New modes of governance and the participatory myth. *West Eur Polit.* 2008b;31(5):874–895. 10.1080/01402380802234540

[ref-33] Sorensen JME Würtzenfeld M Hansen MP : Explaining the deadlock of the European Social Dialogue: negotiating in the shadow of hierarchy. *J Public Policy.* 2022;42(2):323–342. 10.1017/S0143814X21000209

[ref-34] Sorensen TB Dumay X : The European Sectoral Social Dialogue in Education and the strengthening of the European Union's policy regime in education and employment. *J Educ Work.* 2023;36(7–8):542–562. 10.1080/13639080.2023.2275767

[ref-35] Spyropoulos G : Labour law and labour relations in tomorrow's social Europe. *Int Labour Rev.* 1990;129(6):733–750. Reference Source

[ref-36] Velluti S : The European Social Dialogue as a source of EU legal acts following EPSU: collective bargaining and industrial relations get lost in translation. *Common Market Law Rev.* 2022;59(3):871–888. 10.54648/cola2022055

[ref-37] Verbruggen P : Does co-regulation strengthen EU legitimacy? *Eur Law J.* 2009;15(4):425–441. 10.1111/j.1468-0386.2009.00471.x

[ref-38] Zeitlin J Vanhercke B : Socialising the European Semester: EU social and economic policy coordination in crisis and beyond. *J Eur Public Policy.* 2018;25(2):149–174. 10.1080/13501763.2017.1363269

