# Supplementary material for: Revisiting European social dialogue: A systematic literature review
Source: Open Res Eur. 2025 Oct 10;5:309. [Version 1] doi: 10.12688/openreseurope.21020.1 (PMC12701336; doi:10.12688/openreseurope.21020.1)
Supplement: Peer review history

# Reviewer report: Reviewer response for version 1

**DOI:** 10.21956/openreseurope.22739.r62610

**Published:** 2025-12-13

**Authors:** Jan Czarzasty

**Affiliations:** 1 SGH Warsaw School of Economics, Warsaw, Poland

The report is a very valuable contribution to the research on social dialogue in Europe, in general, and on European Social Dialogue (ESD), in particular. As far as the declared objective is concerned (that is, providing a systematic literature review on the subject), it is successfully accomplished. In methodological terms, the whole process is meticulously described. There is no doubt as to relevance of the study, both on formal and substantial levels. The body of research identified in course of the review and covered by the report is abundant but the number of the studies eventually analysed is limited, as vast majority of sources pre-selected were eventually put aside due to not meeting the selection criteria adopted. My concern is about 13 studies excluded, because they were "published in languages other than English". To my understanding, the PRISMA method per se does not require homogeneity of the data in terms of language in which it is published. Furthermore, the new version (2020, which is followed by the Authors) has been developed *inter alia* to address the technological progress including automated translations, as the project team claim (see: Page et al. 2021) and in principle it is possible to use translating devices or human aid. For that reason, maybe it would be worthwhile reconsidering the 13 non-English studies for inclusion in the analysis? Given that 34 articles are now covered, it would enhance the data range substantially and enhance the results, even if only some of the 13 non-English studies could be brought into analysis.

Are sufficient details of the methods and analysis provided to allow replication by others?

Yes

Are the rationale for, and objectives of, the Systematic Review clearly stated?

Yes

Is the statistical analysis and its interpretation appropriate?

Yes

Are the conclusions drawn adequately supported by the results presented in the review?

Yes

Reviewer Expertise:

industrial relations, social dialogue, economic sociology, comparative political economy

I confirm that I have read this submission and believe that I have an appropriate level of expertise to confirm that it is of an acceptable scientific standard.

## References

[1] Page MJ, McKenzie JE, Bossuyt PM, et al.: The PRISMA 2020 statement: an updated guideline for reporting systematic reviews. BMJ. 2021.10.1136/bmj.n71PMC800592433782057

# Reviewer report: Reviewer response for version 1

**DOI:** 10.21956/openreseurope.22739.r62608

**Published:** 2025-10-23

**Authors:** Marius van Staden

**Affiliations:** 1 University of the Witwatersrand, Johannesburg, South Africa

This systematic literature review examines the European Social Dialogue (ESD), a participatory governance mechanism within the European Union that facilitates collaboration between trade unions, employers, and EU institutions on labour policy matters. Using the PRISMA methodology, the authors reviewed 34 peer-reviewed articles published between 1990 and 2024, sourced from Scopus and Web of Science databases. The review synthesises existing knowledge across six thematic areas: definition, regulatory framework, key actors, relationships between actors, negotiation structures, and outcomes.

 The authors identify several critical findings. First, ESD lacks a universally agreed definition, with competing conceptualisations from the International Labour Organisation, the European Commission, and social partners themselves creating conceptual ambiguity. Second, the regulatory framework has evolved significantly since the Treaty of Rome (1957), with key milestones including the Maastricht Treaty (1992) and Amsterdam Treaty (1997), which formalised social partners' roles under Articles 154 and 155 of the Treaty on the Functioning of the European Union. However, since the early 2000s, there has been a marked shift from binding directives to non-binding, autonomous agreements. Third, the review identifies power imbalances between actors, with employers favouring deregulation whilst trade unions face fragmented interests across member states and lack traditional bargaining tools such as the right to strike. Fourth, the authors document structural complexities arising from the division between cross-sectoral and sectoral dialogue frameworks, with ambiguous competency boundaries. Finally, outcomes analysis reveals that fewer than 2% of sectoral documents constitute agreements, with fewer than 10% expected to have meaningful national impact.

 The authors conclude that whilst ESD represents an important participatory governance mechanism, its effectiveness is constrained by conceptual ambiguity, fragmented regulation, limited enforcement capacity, and structural imbalances. They propose three improvements: promulgating a single directive to clarify ESD's legal framework, establishing regular reporting on autonomous agreements, and creating a European Social Dialogue Transparency and Evaluation Board to monitor implementation.

 The methodology section demonstrates several strengths. The authors clearly specify their databases (Scopus and Web of Science), provide the complete Boolean search string, justify the exclusion of the 'ESD' acronym due to overlap with medical terminology, establish clear inclusion and exclusion criteria, and document the selection process using Covidence software with appropriate PRISMA flow diagram. The numbers at each screening stage are transparently reported, and the authors acknowledge limitations including language restrictions and potential selection bias.

 However, several critical methodological details are insufficiently described, which would impede full replication by other researchers. These deficiencies must be addressed to meet contemporary systematic review standards.

 The authors state that "a reviewer carried out three rounds of screening" with subsequent review by "two additional researchers," but provide no information about how disagreements were resolved or whether screening decisions were made independently. Systematic reviews require transparent reporting of inter-rater agreement. Consider adding a paragraph describing: (a) whether title/abstract screening and full-text screening were conducted independently by at least two reviewers; (b) the process for resolving disagreements (consensus discussion, third reviewer arbitration, etc.); and (c) inter-rater reliability statistics (Cohen's kappa or percentage agreement) for at least a subsample of articles. If screening was not conducted independently, this represents a methodological limitation that should be acknowledged explicitly in the limitations section, with justification for the approach taken.

 The manuscript provides no description of how data were extracted from included studies. Readers cannot determine what information was systematically collected, how it was recorded, or who performed the extraction. Consider adding a "Data Extraction" subsection describing: (a) what data were extracted from each article (author, year, research design, key findings related to definition, regulation, actors, structures, outcomes, etc.); (b) whether extraction was performed by one or multiple reviewers; (c) whether a standardised extraction form was used (if available in the OSF repository, reference it explicitly); and (d) how accuracy of extraction was verified.

 This review makes a valuable contribution to understanding ESD as a governance mechanism. The systematic approach successfully synthesises a dispersed literature, the thematic organisation is logical and comprehensive, and the identification of conceptual ambiguities, power imbalances, and implementation gaps represents important scholarly work. The PRISMA methodology is appropriately applied, the writing is generally clear, and the acknowledgement of AI tool usage demonstrates commendable transparency. The review fills a genuine gap by providing structured synthesis of ESD scholarship.

Are sufficient details of the methods and analysis provided to allow replication by others?

Partly

Are the rationale for, and objectives of, the Systematic Review clearly stated?

Yes

Is the statistical analysis and its interpretation appropriate?

Yes

Are the conclusions drawn adequately supported by the results presented in the review?

Yes

Reviewer Expertise:

Labour and Employment Law.

I confirm that I have read this submission and believe that I have an appropriate level of expertise to confirm that it is of an acceptable scientific standard.
